# AR-A 014418 Used against GSK3beta Downregulates Expression of hnRNPA1 and SF2/ASF Splicing Factors

**DOI:** 10.1155/2014/695325

**Published:** 2014-01-02

**Authors:** Ajay K. Yadav, Vidhi Vashishta, Nidhi Joshi, Pankaj Taneja

**Affiliations:** ^1^Dr. B. R. Ambedkar Center for Biomedical Research, Delhi University, Delhi 110007, India; ^2^Institute of Nuclear Medicine and Allied Sciences, Timarpur, Delhi 110007, India

## Abstract

Glioblastoma is one of the most aggressive forms of primary brain tumors of glial cells, including aberrant regulation of glycogen synthase kinase 3**β** (GSK3**β**) and splicing factors deregulation. Here, we investigate the role of small molecule AR-A014418 and Manzamine A against GSK3 kinase with factual control on splicing regulators. AR-A 014418, 48 hrs posttreatment, caused dose (25–100 **μ**M) dependent inhibition in U373 and U87 cell viability with also inhibition in activating tyrosine phosphorylation of GSK3alpha (Tyr 279) and beta (Tyr 216). Furthermore, inhibition of GSK3 kinase resulted in significant downregulation of splicing factors (SRSF1, SRSF5, PTPB1, and hnRNP) in U87 cells with downregulation of antiapoptotic genes such as BCL2, BCL-xL, Survivin, MCL1, and BMI1. Similarly, downregulation of splicing factors was also observed in U373 glioma cell after using SiRNA against AKT and GSK3beta kinase. In addition, potential roles of AR-A014418 in downregulation of splicing factors were reflected with decrease in Anxa7 (VA) variant and increase in Anxa7 WT tumor suppressor transcript and protein. The above results suggest that inhibition of GSK3beta kinase activation could be the beneficial strategy to inhibit the occurrence of alternative cancer escape pathway via downregulating the expression of splicing regulators as well as apoptosis.

## 1. Introduction

Glioblastoma remains challenging to us being a devastating aggressive brain tumor. Patients median survival is found to be around 15 months and 10% less will survive 5 years after diagnosis [1, 2]. GBM's heterogeneous genome wide landscape and resistance against multimodal therapy make this even more challenging to treat or to at least extend the survival of patient beyond expectancy. Although a lot of progress is being made to revive the multigene targeted approaches to restrict the tumor growth, it is going to be limited due to its biological complexity and tumor heterogeneity [3]. Moreover finding a better target for GBM could be possible by studying the regulation of splicing factors involved in evolving alternative splicing event with diversified encoded protein product in cancer to escape apoptotic pathway. Cooperative and competitive association between splicing factor enhancer and silencer is under exploration area; its functional consequences lead to misregulated alternative splice variant expression which could affect biology of survival signalling in various scales of function such as mRNA stability, oncogenic gain, and loss in tumor suppressor function. However, few of splicing factors such as PTBP1 (polypyrimidine tract binding protein), SRSF1 (SR protein), and hnRNPs are found to be aberrantly overexpressed in breast cancer, gliomas, and so forth [4, 5]. Out of these in detail, SRSF1 found directly modulates the expression of tumourigenic the tyrosine kinase receptor (RON), is associated with shorter survival and poorPrognosis in GBM's patient [6].

Since splicing factors such as SF2/ASF1 and hnRNPs are overexpressed in various cancers with constitutively active growth survival signals, the connecting link between initiating EGFR (epidermal growth factor receptor)—Akt kinase—GSK3beta kinase survival signaling pathways with regulation of splicing factors is barely been studied. However, few of the earlier reports showed p38 kinase mediated upregulation of nuclear hnRNPA1 [7] in senescent fibroblast with inhibition of nucleocytoplasmic shuttling, and recently [8] a link between Akt kinase and hnRNPs regulation has been hypothesized.

In the current set of investigations, we are studying the expression of splicing factors which are the major deregulated family proteins in maneuvering the alternative splicing event to escape apoptotic pathway, potentially could be explained after using small molecular inhibitors against Akt kinase and GSK3beta with ability to restrict the occurrence of alternative pathway either by reversing or decreases the occurrence of alternative splicing, and will possibly help in restricting the tumor genetic heterogeneity.

## 2. Results

### 2.1. Cell Viability Assay after Inhibiting GSK3beta and Akt Kinase

**Figure 1:**
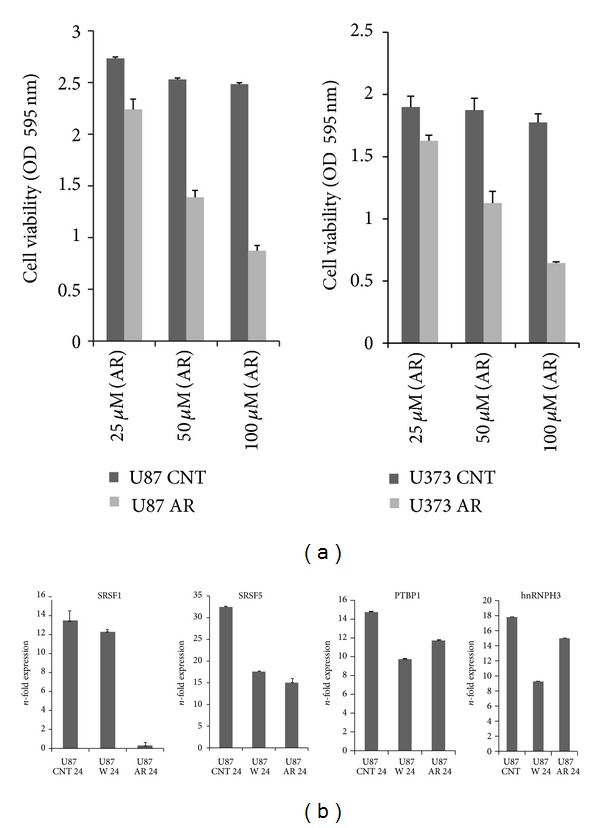
Cell viability assay and qRTPCR assay for splicing factors: (a) cell viability assay for dose dependent response of U87 and U373 treated with AR-A 014418 for 48 hrs; (b) qRT-PCR of splicing factors (SRSF1, SRSF5, PTBP1, and hnRNPH3) in U87 glioma cell line after treating cells with Wortmannin and AR-A 014418 in different experiments for 24 hrs. Means ± S.E.; *n* = 5; *P* ≤ 0.05.

Tumor reoccurrence and resistance are of wide concern to study in detail the major deregulated multitude signalling pathways. Assessing cell viability is one of the parameters to study mitochondrial function and its attenuation using small molecule against GSK3beta kinase, eventually inducing apoptotic signalling pathway. Hence, to study the role of GSK3beta in cell viability, we have chosen dose dependent temporal kinetics of small molecule AR-A 014418 and Manzamine A against GSK3 kinase. We found that 50 *μ*M AR-A 014418 is required to inhibit the (>50%) glioma cell viability (Figure 1(a)).

### 2.2. Regulation of Splicing Factor Using GSK3beta and Akt Kinase Inhibitors

**Figure 2:**
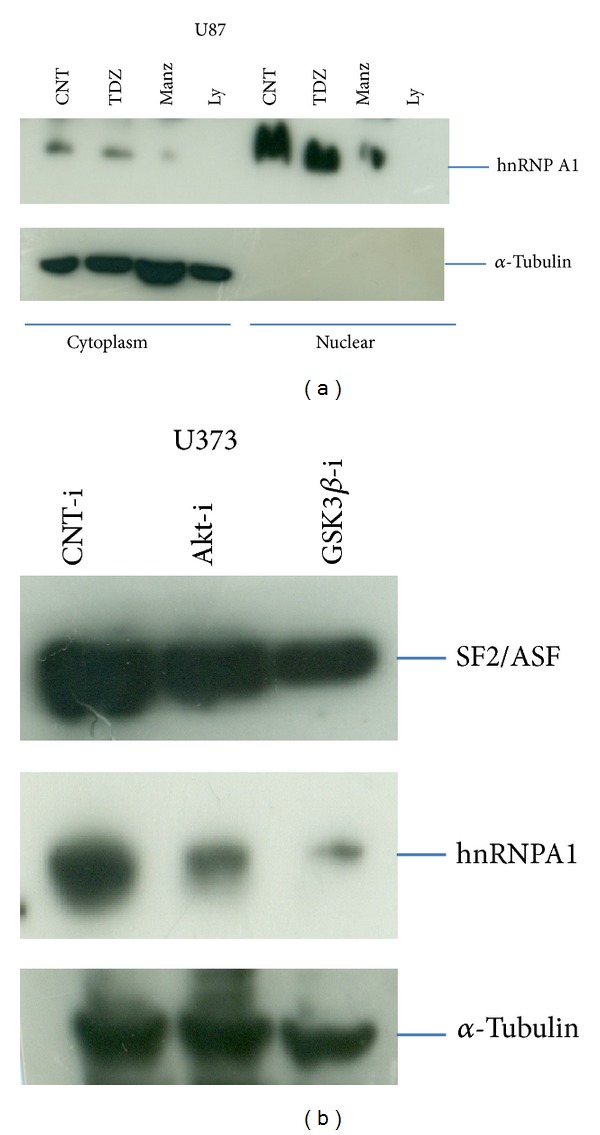
Splicing factor regulation. (a) U87 glioma cell lines were treated with TDZ8 (20 *μ*M), Manzamine A (2 *μ*M), and LY294002 (10 *μ*M) for 24 hrs, followed by cytoplasmic and nuclear extraction, studied hnRNPA1 downregulation. Results were evaluated from multiple different experiments. (b) U373 glioma cells were transfected with Akt RNAi and GSK3beta RNAi compared with CNT RNAi, studied SF2/ASF1 and hnRNPA1 downregulation.

Since deregulated expressions of splicing factors such as PTBP1, SF2/ASF (SRSF1, SRSF5), and hnRNPs are the major cause that interfere with the homeostatic signaling proteins by inducing translational expression of diversified alternative splice variant, with antiapoptotic gain or apoptotic loss in advancement of tumorigenicity. Functional significance of GSK3beta in arresting gliomagenesis, though downregulating the overexpression of splicing factor using inhibitors against GSK3beta, were evaluated (Figure 1(b)) compared with Wortmanin (Akt kinase inhibitor). Similarly in one of the experiment downregulation of hnRNPA1 in nuclear compartment of U87 glioma cells after treating with small molecule against GSK3beta and Akt kinase, Moreover, downregulation of hnRNPA1 and SF2/ASF1 in U373 glioma cell were also observed after Akt RNAi and GSK3beta RNAi (Figure 2), this further opening the avenue of multifunctional role of GSK3beta including regulation of splicing factor expression by which could restrict the origination of alternative genetic splice variant or alternative apoptotic escape pathway.

### 2.3. Regulation of Apoptotic Regulators

**Figure 3:**
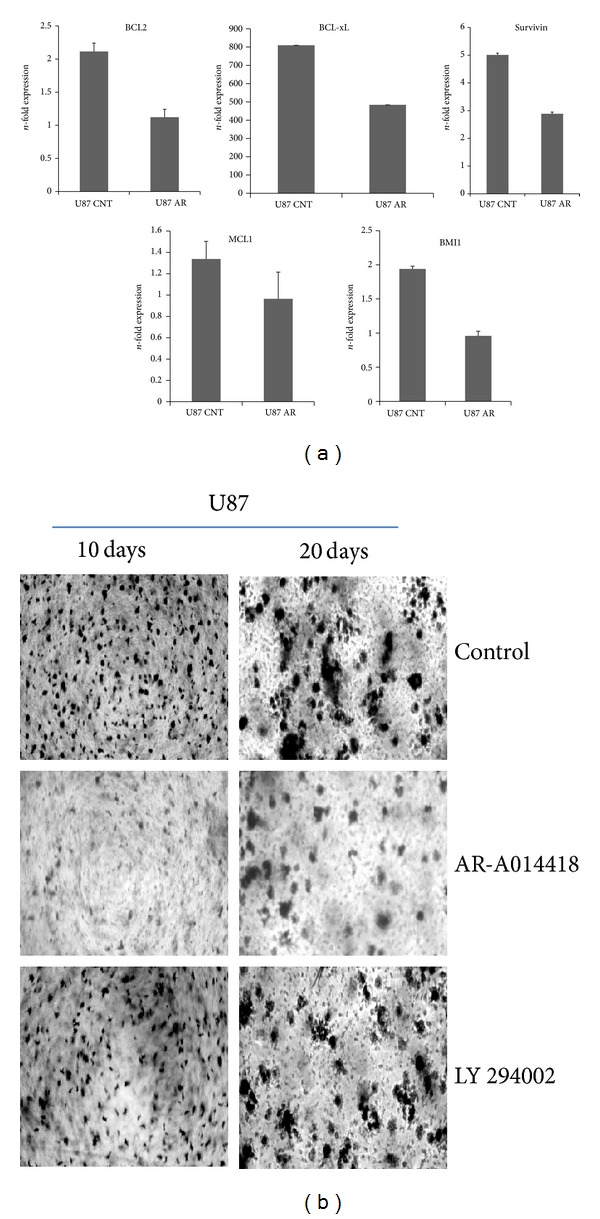
(a) qRTPCR analysis of BCL2, BCL-xL, Survivin, MCL1, and BMI1 in glioma cell lines treated or untreated with AR-A 014418 (50 *μ*M) for 24 hrs. Means ± S.E.; *n* = 5; *P* ≤ 0.05. (b) Soft agar assay to estimate the tumorigenic propensity of U87 cells in presence of AR-A 014418 (50 *μ*M) and LY294002 (10 *μ*M), observed for 10 days and 20 days.

Similarly genetic expressions of antiapoptotic regulators such as BCL-xL, Survivin, and MCL1 are significantly downregulated using specific small molecule inhibitors against GSK3beta kinase, as earlier shown by the other group using small molecule used against Akt kinase [9]. In addition, BMI1 (polycomb protein family member) involved in stem cell self-renewal and cancer stem cell maintenance [10] is also significantly downregulated (Figure 3(a)), which seems to reflect in tumorigenic potential of U87 cells in the presence of AR-A 014418 (GSK3beta inhibitor) but not with Akt inhibitor studied using protocol of soft agar assay (Figure 3(b)), suggesting that the tyrosine phosphorylation of GSK3beta kinase is critical in regulating the colony formation (Figure 5(c)).

### 2.4. Glioma Cell Colonies Formation

GSK3beta inhibition, using AR-A 014418 and Akt kinase inhibitor Ly294002 compared with control (untreated), showed decrease in evolution of U87 glioma cells originated colonies on soft agar plate assay suggesting potential involvement of GSK3beta compared to Akt kinase (Figure 3(b)).

### 2.5. Regulation of GSK3beta Mediated Signaling Pathway

**Figure 4:**
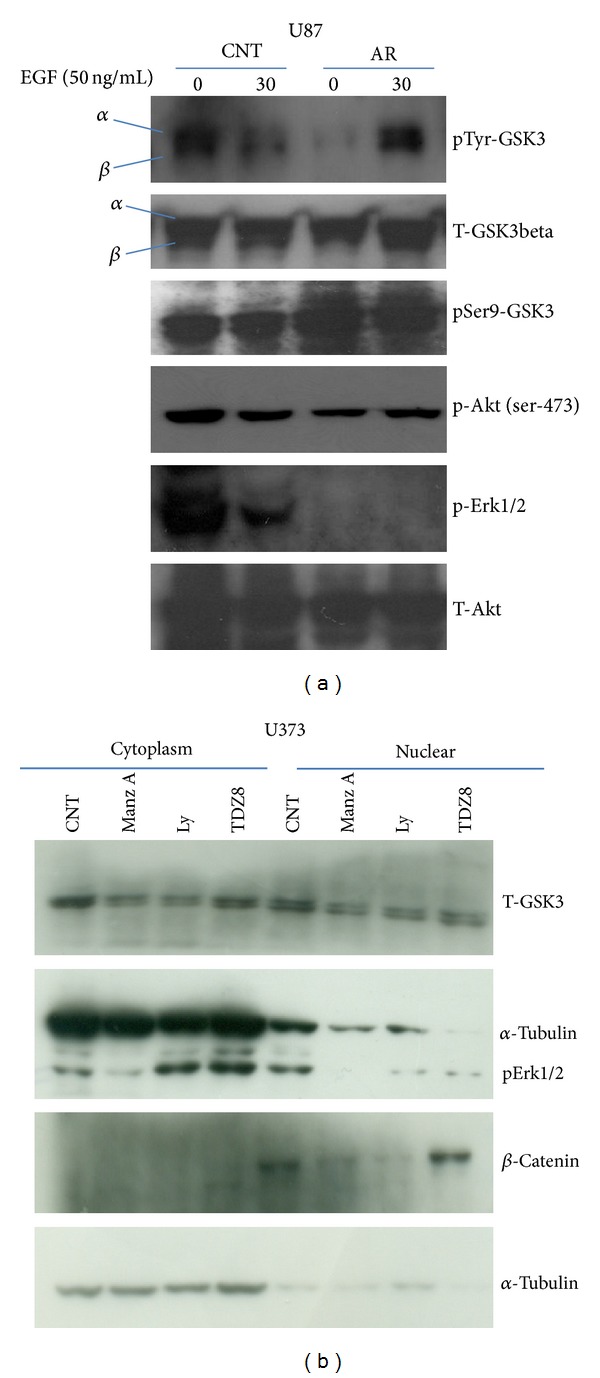
U87 and U373 glioma cell lines were grown and plated day before, incubated with small molecule inhibitors against GSK3beta kinase (AR-A 014418, Manzamine A, and TDZ-8), Akt kinase inhibitor (LY294002 and Wortmanin), and Bortezomib (BR) for 24 hrs. (a) EGFR ligand dependent regulation of activating tyrosine phosphorylation of GSK3 kinase and downregulation of ERK1/2 pathway, after inhibiting the tyrosine phosphorylation of GSK3 kinase using AR-A 014418 for 24 hrs. (b) Differential localization of GSK3 kinase, beta catenin, and p-ERK1/2 was studied in cytoplasmic and nuclear fraction isolated from treated U373 glioma cell line. All experiments were repeated multiple times.

**Figure 5:**
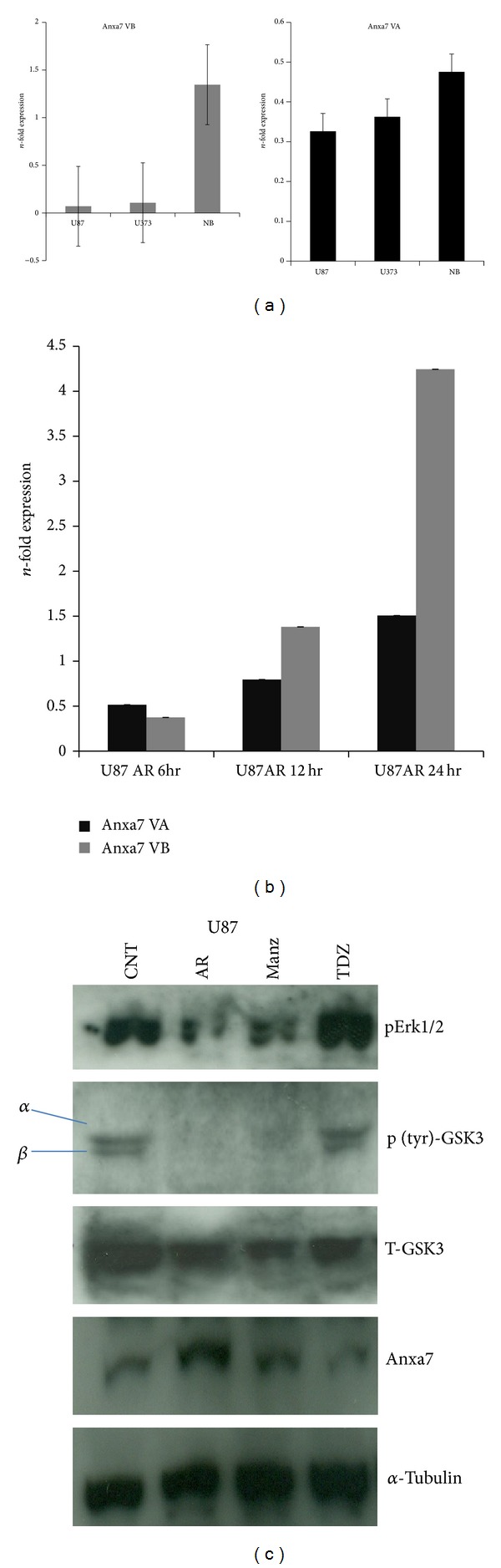
Anxa7 splicing in U87 glioma. (a) Estimation of Anxa7 VA (variant) and Anxa7 VB (WT) in U373 and U87 glioma cells compared with Normal Brain cDNA. (b) Temporal kinetics of Anxa7 splicing (6, 12, and 24 hrs) in presence of AR-A 014418 (GSK3beta kinase inhibitor), Means ± S.E.; *n* = 5; *P* ≤ 0.05. (c) Small molecule against GSK3 kinase used to inhibit activated Tyrosine phosphorylated GSK3 kinase in U87 cells incubated for 24 hrs observed restoration of Annexin 7 tumor suppressor protein.

Inhibition of activating tyrosine phosphorylation of GSK3alpha (Tyr 279) and -beta (Tyr 216) observed after using GSK3beta inhibitor AR-A 014418 (50 *μ*M) and Manzamine A (2 *μ*M) but not by using TDZD-8 (20 *μ*M) and Ly294002 (10 *μ*M) suggests comparative high antitumorigenic potential of AR-A 014418 and Manzamine A (Figures 4(a) and 5(c)). Similarly, translocations of GSK3 kinase, beta catenin, and pERK1/2 to the nucleus were significantly affected after using small molecule Manzamine A, Ly294002, and TDZD-8 (Figure 4(b)). Downregulation of GSK3beta activating tyrosine phosphorylation without change in Ser9-GSK3beta phosphorylation negates the involvement of Akt kinase mediated downstream GSK3beta inactivation. However, involvement of potential Akt kinase independent of EGFR ligand induced GSK3beta mediated MAP kinase regulation has been observed (Figure 4(a)). In addition, upregulation of Annexin 7 protein on GSK3beta inhibition (Figure 5(c)) was found very interesting to explore epidermal growth factor receptor (EGFR) regulation [11].

### 2.6. GSK3beta Inhibition Induced Reversal in Anxa7 Alternative Splicing

Anxa7 is a Ca2+/phospholipid binding protein involved in membrane fusion and tumor suppressor protein [12].

Earlier we have shown that Anxa7 is a EGFR negative regulator; its allelic loss has been depicted with enhanced glioma survival signaling pathway [11, 13].

Here we have observed splicing deregulation with elevated Anxa7 VA (variant) transcript with loss in Anxa7 VB (WT) in comparison with Normal Brain cDNA Clontech company in U87 and U373 glioma cells (Figure 5(a)). However, inhibition of GSK3beta activation in presence of AR-A 014418 treated for different time points (0, 6, 12, and 24 hrs), showed gradual inhibition in alternative splicing of Anxa7 with increase in Anxa7 VB (WT) in between 6 hrs and 12 hrs, with increase in Anxa7 VB transcript in time dependent manner (Figure 5(b)). In addition restoration of Annexin 7 protein on GSK3beta inhibition (Figure 5(c)) was found very interesting to explore epidermal growth factor receptor (EGFR) regulation.

## 3. Discussion

GSK3 was been accepted and viewed as nodal regulator in varied multiple cellular signaling pathway; moreover its role is widely dispersed including cell structure, metabolism, and cell survival. Inhibition of GSK3beta has been demonstrated largely in treating neurodegenerative diseases [14]; however, involvement of GSK3beta in cancer progression varies among different cancers.

Differential expression and regulation of GS3*β* in cancer appear to be tissue specific and involved in inducing molecular genetic heterogeneity, as certain forms of cancer showed high expression of active GSK3*β* [15]; however, other cancer tissues harbor low levels. It was suggested that high levels of nuclear GSK3*β* in pancreatic cells were associated with dedifferentiation and NF-kB mediated survival [16]. GSK3*β* activity also appears to be important for leukemic cell growth, as the inhibition of GSK3*β* led to an induction of apoptosis in leukemic cells and also showed that GSK3*β* has proapoptotic role in lung cancer by downregulating Survivin activity [17], whereas GSK3beta kinase inhibition in glioma cells induces proapoptotic affect [18, 19].

Additionally the membrane associated growth factor receptor (GFR) induced survival signaling pathway, and recruitment of oncogenic signals for nuclear transcriptional regulation could be aberrant due to aberrant activity of GSK3beta as demonstrated earlier [20], while involvement of intermediary survival kinases with apparent deregulation of splicing factors is sparsely understood. However, deregulatory behavior of splicing factors with apparent rise in alternative splicing is now the causing hallmark to investigate further the alternative apoptotic escape pathways, Nonetheless possibly by aligning the functional role of Growth factor survival kinases with deregulation of splicing regulators! Moreover, activated ERK1/2 kinase driven GSK3beta priming is one of the essential molecular modulating events to regulate [21] the downstream signaling event. Hence, to explore the inhibition of GSK3beta kinase with significant repression of tumorigenic potential in glioma cell line [22], our study showed that inhibition of GSK3beta Tyrosine-216 phosphorylation activity without change in inactivated Ser9 phosphorylation leads to downregulation of the expression of GSK3beta with loss of ERK1/2 phosphorylation. To extend GSK3beta kinase activity forward in term of regulation of splicing factors such as PTBP1, SF2/ASF and hnRNPs, which are the leading cause for the origination of alternative splice encoded proteins are of wide concern, such as caspase 9 variant origination involve in occurrence of alternative escape apoptotic pathways [23] found Akt kinase dependent [8]. Interestingly, small molecule against GSK3beta mediated additional downregulation of splicing factors such as PTBP1, hnRNPA1, and SF2/ASF which have all been observed first time by us, along with decrease in antiapoptotic regulators such as MCL1, Survivin, and BMI1 as shown earlier [9]. Moreover, small molecule mediated intervention appears to be supportive experimental observation using knock-down approach where knocking down Akt or GSK3beta kinase leads to downregulation of hnRNPA1 and SF2/ASF (SRSF1), suggesting the involvement of partial Akt kinase and GSK3beta kinase in splicing factor downregulation, which is demonstrated further using small molecule (therapeutic) approach against GSK3beta such as Manzamine A and AR-A 014418. Multiple roles of hnRNPs complex on Anxa7 tumor suppressor protein have been demonstrated as potential inducer of Anxa7 alternative splice variants [24] in prostate cancer. Moreover, loss of Anxa7 tumor suppressor expression with gain in epidermal growth factor receptor protein induced survival signaling pathway and reported earlier, demonstrated to be a high risk factor in evaluating the survival of glioblastoma patient [11, 13]. We, therefore, investigated Anxa7 alternative splicing using small molecule against GSK3beta and invariably demonstrated inhibition in Anxa7 variant with increase in transcript of Anxa7 WT gene. Similarly, Anxa7 protein restoration using small molecule against GSK3beta was found to be an additional advantage along with downregulation of MCL1 and Survivin in glioma cells, which is supported by the earlier group; the relevance of radiation induced glioma's sensitization after downregulation of MCL1 and Survivin using Akt kinase inhibitor in EGFR context [25, 26].

In conclusion GSK3beta inhibition results in decrease in splicing factors such as PTBP1, SF2/ASF, and hnRNPA1, decrease in antiapoptotic regulators, and increase in Anxa7. Small molecules, such as Manzamine A and AR-A 014418 [27], used against GSK3beta kinase should be further checked to study widely the alternative splicing event. These results clearly indicate the functional significance of Gs3kbeta in arresting gliomagenesis by these inhibitors. Moreover future targeted drug therapeutic strategies can be designed based on the findings of these nodal GSK3beta kinase regulated pathways.

## 4. Materials and Methods

### 4.1. Cell Culture and Reagents

U87 and U373 human glioma (astrocytoma) cells were propagated by Dulbecco's modified Eagle's medium (DMEM) containing 10% fetal bovine serum (Invitrogen, Carlsbad, CA, USA), 100 Units/mL penicillin, and 100 *μ*g/mL streptomycin. Cells were seeded in 100 mm plates and incubated overnight, followed by treatment with LY294002 or Wortmanin (Akt kinase inhibitor), AR-A 014418 or Manzamine A or TDZD8 (GSK3beta inhibitor), and proteasome inhibitor (Bortezomib) at various concentrations for 48 hrs. Normal Brain RNA was purchased from Clonotech company.

### 4.2. Quantitative Real-Time PCR

Quantitative real-time polymerase chain reaction (PCR) was performed using standard procedures to determine relative mRNA transcript levels. Total RNA was extracted using TRIzol Reagent (Invitrogen, Carlsbad, CA, USA), and first strand cDNA was synthesized using random hexamer and superscript II (Invitrogen, Carlsbad, CA, USA). Quantitative real-time PCR was performed using MESA Green qPCR MasterMix Plus for SYBR Assay in 7300 thermocycler (Applied Biosystems). Thermal cycling consisted of a warm-up step of 2 min at 50°C and initial denaturation at 95°C for 10 m, followed by 40 cycles of each polymerase chain reaction (PCR) step: 95°C for 15 s and 60°C for 1 m. All primers used for real-time PCR analysis were synthesized by IDT (Integrated DNA Technologies). The specificity of each primer pair was confirmed by melting curve analysis and running amplified sample in agarose-gel electrophoresis. GAPDH was used as internal control. List of various gene primers (5′ to 3′) was enlisted in Table 1.

### 4.3. Cell Viability Assay

Cell viability was assessed using the MTT colorimetric assay, based on the ability of viable cells to reduce yellow MTT (3-(4-5-dimethylthiazol-2-yl)-2,5-diphenyltetrazolium bromide, a tetrazole) to purple formazan product which is largely impermeable to cell membranes, thus resulting in its accumulation within living cells. Solubilization of the cells results in the liberation of the purple product which can be detected using a colorimetric measurement. Glioma cells were cultured in 96-well microlitre plates for various time periods in the presence of inhibitors. After incubation, cells were observed under a contrast phase microscope before adding MTT solution, prepared fresh as 5 mg/mL in DMEM, filtered through a 0.22-*μ*m filter, and kept for 5 min at 37°C. MTT solution (100 *μ*L) was added to each well, and the plates were incubated in the dark for 4 h at 37°C, and subsequently solubilized in DMSO, the extent of reduction of MTT was quantified by absorbance measurement at 595 nm. Experimental groups were performed in at least triplicate.

### 4.4. Soft Agar Assay

U87 glioma cells were grown and trypsinized, counted, suspended in 0.2% agar in 20% fetal bovine serum DMEM, and equally (10^5^ cell number) plated in duplicate on 60 mm dishes containing 0.5% agarose bed. Growing DMEM medium containing LY294002 (Akt kinase inhibitor), AR-A 014418, or Manzamine A was added next day. Colonies were observed and counted at 3 different sites at various time points (days 10 and 20), stained for viability with 3 mg/mL 3-(4,5-dimethylthiazol-2-yl)-2,5-diphenyltetrazolium bromide (MTT) for 4 hours, and visualized under standard light microscope. Colony formation indicates that individual cells develop into cell clones that are identified as single colonies.

### 4.5. SiRNA Transfection

Glioma cells were transfected a day after plating of glioma cells with SiRNA against Akt and GSK3beta kinase compared with CNT-i (Santa Cruz Inc.) as per Santa Cruz Inc. protocol. Lysates were made after 48 hrs of transfections in Triton X-100 buffer.

### 4.6. Protein Extraction and Immunoblotting

U87 and U373 glioma cell lysates were prepared in 0.1% Triton buffer containing protease and phosphatase inhibitors (Sigma-Alrich) and also were prepared cytoplasmic and nuclear extracts using cell compartment isolation kit (Millipore). Further total protein was quantitated using the BCA Protein Assay Kit (Thermo Scientific, Rockford, IL). Blots were exposed to anti-GSK3 (Millipore), anti-phosphotyrosine GSK3 (Millipore), anti-phospho-ERK1/2 (Thr202/Tyr204) (Millipore), anti-*α*-tubulin, and anti-beta-catenin (Millipore) and recognized by an HRP-conjugated horse anti-mouse secondary antibody (Santa Cruz Inc); antibodies to detect phospho-AKT1/2/3 (Ser473), total Akt1, hnRNPA1, and Anxa7 (Santa cruz Inc) SF2/ASF1 (Santa Cruz Inc) were recognized by an HRP-conjugated goat anti-rabbit secondary antibody (Santa Cruz Inc.). Enhanced chemiluminescent reagent (GE Healthcare) was added to the membranes according to the manufacturer's protocol and visualized by autoradiography.

### 4.7. Statistical Analysis

Experimental results have been expressed as the mean ± standard error (S.E.). Statistical differences of data were assessed by Student's-*t*-test. *P* values lower than 0.05 were considered significant.

## Figures and Tables

**Table 1:** List of primers used to estimate the quantitative genetic transcription after incubating with different kinase specific inhibitors.

S. number	Gene name	Forward primer (5′ to 3′)	Reverse primer (5′ to 3′)
1	GAPDH	5′ACCACAGTCCATGCCATCAC 3′	5′TCCACCACCCTGTTGCTGTA 3′
2	SF2/ASF1	5′CACTGGTGTCGTGGAGTTTG 3′	5′TCCTGCTGTTGCTTCTGCTA 3′
3	hnRNPH3	5′ TGAGGCTAGTGATGGGACAG 3′	5′ TGTTTCCCCAGAGCATTTTC 3′
4	PTBP1	5′ ACGGACCGTTTATCATGAGC 3′	5′AGCATCAGGAGGTTGGTGAC 3′
5	Survivin	5′ TTGCTCCTGCACCCCAGAGC 3′	5′ AGGCTCAGCGTAAGGCAGCC 3′
6	BCL-xL	5′ TCTCCTTTGGCGGGGCACTG 3′	5′ TCCACAAAAGTGTCCCAGCCGCC 3′
7	BCL2	5′CTCTCGTCGCTACCGTCGCG3′	5′AGGCATCCCAGCCTCCGTTATCC 3′
8	MCL1	5′ TGGAGGTGAACCCGACT TCCATG 3′	5′ TGGGGCTGGCTTGAGGTTCTCAA 3′
9	BMI1	5′GGAGACCAGCAAGTATTGTCCTATTTG3′	5′CTTACGATGCCCAGCAGCAATG 3′
10	Anxa7 VB (WT)	5′TCTCCTGTTTCTTTGGATTATAG 3′	5′ACCCTTCATTGCCTTACG3′
11	Anxa7 VA	5′CCCTAGTCAGCCTGCCACAGT3′	5′ACCCTTCATTGCCTTACG3′

## Abbreviations

hnRNP: Heterogeneous ribonucleoprotein particle
SRSF or SF2/ASF: Serine/arginine-rich splicing factor 1
EGFR: Epidermal growth factor receptor
BCL-xL: B-cell lymphoma-extra large
BMI-1: B lymphoma Mo-MLV insertion region 1 homolog
MCL1: Myeloid cell leukemia sequence 1.
